# Preparation, Characterization, and In Vitro Anticancer Activity Evaluation of Broccoli-Derived Extracellular Vesicle-Coated Astaxanthin Nanoparticles

**DOI:** 10.3390/molecules27123955

**Published:** 2022-06-20

**Authors:** Chunmei Li, Qi Song, Xialian Yin, Ruilong Song, Gang Chen

**Affiliations:** 1 College of Food Science and Engineering, Yangzhou University, Yangzhou 225009, China; licm@yzu.edu.cn (C.L.); 18362087256@163.com (Q.S.); yxl1904186721@163.com (X.Y.); 2 Institute of Comparative Medicine, College of Veterinary Medicine, Yangzhou University, Yangzhou 225009, China; rlsong@yzu.edu.cn; 3 School of Rehabilitation Science and Engineering, University of Health and Rehabilitation Sciences, Qingdao 266024, China

**Keywords:** extracellular vesicles, astaxanthin, broccoli, preparation, anticancer activity

## Abstract

Astaxanthin (AST) is a type of ketone carotenoid having significant antioxidation and anticancer abilities. However, its application is limited due to its low stability and bioavailability. In our study, poly (lactic-co-glycolic acid) (PLGA)-encapsulated AST (AST@PLGA) nanoparticles were prepared by emulsion solvent evaporation method and then further processed by ultrasound with broccoli-derived extracellular vesicles (BEVs), thereby evolving as BEV-coated AST@PLGA nanoparticles (AST@PLGA@BEVs). The preparation process and methods were optimized by three factors and three levels of response surface method to increase drug loading (DL). After optimization, the DL was increased to 6.824%, and the size, polydispersity index, and zeta potential of AST@PLGA@BEVs reached 191.60 ± 2.23 nm, 0.166, and −15.85 ± 0.92 mV, respectively. Moreover, AST@PLGA@BEVs exhibited more notable anticancer activity than AST in vitro. Collectively, these results indicate that the method of loading AST in broccoli-derived EVs is feasible and has important significance for the further development and utilization of AST as a functional food.

## 1. Introduction

Astaxanthin (AST) is a pigment synthesized from lycopene, part of the lutein family, and an oxidized carotenoid derivative [1]. It is found in many of our favorite shellfish, salmon, trout, red snapper, shrimp, lobster, and eggs [2]. AST has a number of important biological functions, including the prevention of oxidation in unsaturated fatty acids, ultraviolet radiation resistance, immune response, pigmentation, information transmission, and reproductive improvement [3]. Among these functions, one of the most important properties of AST is its antioxidant effect. Studies have shown that its antioxidant properties exceed those of β-carotene and α-tocopherol. Given its outstanding antioxidant activity, many researchers believe that AST can protect the body against many oxidative-related common diseases, including cardiovascular diseases, different types of cancer, and some diseases of the immune system. Thus far, most of the applications of AST are for human nutrition and health [4,5]. Chen et al. reported that AST can inhibit the growth, migration, and invasion of melanoma A375/A2058 cells and downregulate the expression of MMP-1, -2, and -9 in a dose-dependent manner [6]. AST has potential physiologic functions, such as relieving oxidative stress, anti-tumor, anti-inflammatory, anti-ultraviolet light oxidation, and anti-Helicobacter pylori infection [7]. However, the 11 double-conjugated links in the AST structure restrict its stability, leading the volatile AST to be affected by unfavorable environmental factors, including heat, radiation, oxygen, and alkaline or acidic solutions [8]. Moreover, its high lipophilicity and heat resistance limit its antioxidant effect in biomedical applications.

In recent years, there has been a great growth in the research of nanopharmaceutics. Nanopharmaceutics have advantages in morphology and size and can improve the performance and stability of drugs. Because it can use different types of raw materials to ensure specific properties, nanotechnology has received widespread attention in the pharmaceutical and food fields. In the food industries, nanotechnology can be used for preparing nanonutraceuticals, which can improve the shortcomings and improve the performance of nutritional components. However, relevant systems and good manufacturing practices are still needed to ensure the quality and safety of the final products [9,10]. Nowadays, nanotechnologies have been widely used to solve the AST instability issue. For instance, Rodriguez–Ruiz, et al. used sunflower oil as a green liquid lipid processed to synthesize a nanostructured lipid carrier (NLCs) loaded with natural AST. The antioxidant activity was then measured by the method of α-tocopherol-equivalent antioxidant capacity and compared with natural AST and α-tocopherol. The AST load studies showed that the recovery rate of AST from AST-loaded NLCs exceeded to 90%. The result indicated that the preparation could stabilize the AST molecule and maintain or even improve its antioxidant capacity [11].

Protein secretion is important for eukaryotic cells. Unconventional protein secretion can secrete proteins without signal peptides or other cytoplasmic substances into the extracellular space, which may be responsible for extracellular vesicle (EV) formation [12]. In various diameters, EVs can be divided into exosomes, microvesicles, and apoptotic bodies. Exosomes are 40–100 nm in diameter, whereas microbubbles and apoptotic bubbles are 100–350 and 500–1000 nm in diameter, respectively [13]. Many studies on EVs focus on mammals as the main direction. Recently, plants were discovered to secrete EVs which play a major role in plant cell communication and immune regulation. They can protect plants against the invasion of pathogenic bacteria [14]. Plant-derived exosome-like nanoparticles (PDENs) can be taken up by gut macrophages and interspecifically communicate by inducing various cytokines [15]. Moreover, the extraction rate for PDENs is much higher than in mammalian cells [16], illustrating their economic potential as a nanoplant [17]. EVs transmit payloads to other species for cross-border communication. Their characteristics and transport capabilities improve the applicability of pharmaceutical transportation [18]. PDENs and synthetic nanoparticles (such as liposomes and micelles) have common basic characteristics, including a similar lipid bilayer structure and the ability to carry hydrophilic or hydrophobic cargos [19]. However, EVs are superior to synthetic nanoparticles in terms of nontoxicity, low immunogenicity, increased cellular absorption, greater stability in the gastrointestinal tract, and specific targeting capability [20]. For example, PDENs have 10 times more specificity for tumor targeting than liposomes [21], less potential for unnecessary gene or protein transfer prior to clinical application than nanoparticles derived from mammalian or bacterial cells, and almost no harmful immunogenic reactions [22]. Furthermore, different from other delivery vehicles, PDENs have a controlled liquid phase film, which promotes their ability to resist surfactant dissolution. PDENs have also proven resistant to digestive enzymes (such as intestinal trypsin, pepsin, and bile extract). This important feature helps them protect the drug from resolving in the stomach or intestinal environment, leading to safe drug administration in the desired colon [23].

Currently, the two major obstacles to cancer treatment are low specificity and high toxicity. To tackle these great challenges, researchers are striving to develop a range of new drug delivery systems. For example, scientists have successfully used ginger-derived PDENs to prepare nanocarriers to deliver doxorubicin to colon cancer cells. These nanocarriers internalize the colon-26 tumor model cells and inhibit their growth. Interestingly, the significant antitumor effect is not only derived from the anticancer activity of Adriamycin but also from the inhibitory effect of PDENs on oxidative stress [24]. Reports showed that PDENs have no toxicity and good biocompatibility and can be produced economically on a large scale. Compared with synthetic liposomes, they are superior to liposomes in biocompatibility, safety, apoptosis, cell substrate impedance, and drug release [25].

In our study, poly (lactic-co-glycolic acid) (PLGA)-encapsulated AST (AST@PLGA) nanoparticles were prepared by emulsion solvent volatilization method, and broccoli-derived extracellular vesicle (BEV)-coated AST@PLGA (AST@PLGA@BEVs) nanoparticles were prepared by ultrasound. The response surface methodology was used to optimize the drug loading. After characterizing the optimized nanoparticles, the in vitro anticancer activities of AST, AST@PLGA, and AST@PLGA@BEVs were contrasted and analyzed by HT-29 cells.

## 2. Materials and Methods

### 2.1. Materials

PLGA (lactide:glycolide 50:50; Mw 38,000–54,000) and Poly vinyl alcohol (PVA, P875084) were purchased from MACKLIN (Shanghai, China). Astaxanthin (AST, SML0982) was purchased from Sigma-Aldrich (St Louis, MO, USA). MTT Cell Proliferation and Cytotoxicity Assay Kits were purchased from Beyotime (Shanghai, China). Dichloromethane and Ethanol were purchased from SINOPHARM (Beijing, China).

### 2.2. Isolation and Purification of BEVs

Broccoli (1000 g) was washed with water, smashed, and filtered to prepare broccoli sap. The broccoli sap was isolated using differential centrifugation at 500× *g* for 10 min, 2000× *g* for 20 min, 5000× *g* for 30 min, and 10,000× *g* for 1 h at 4 °C. The supernatant used ultrahigh speed centrifugation at 150,000× *g* for 2.5 h at 4 °C [26]. The pellet was resuspended in phosphate-buffered saline (PBS).

### 2.3. Preparation of the Nanoparticles Loaded with AST

The AST@PLGA nanoparticles were prepared by the emulsion solvent volatilization method [27]. The weighed AST and PLGA were dissolved in 1 mL of dichloromethane and then completely dissolved by ultrasound as an organic phase. The organic phase was slowly added to the PVA solution drop by drop, and then the emulsion was obtained by ultrasound. The emulsion was stirred on a magnetic stirrer for 3–4 h at room temperature and after centrifugation at 14,000 rpm for 40 min at 4 °C. The pellet was resuspended in PBS. Finally, BEVs and AST@PLGA were sonicated for 5 min in a water bath at 40% power.

### 2.4. Drug Loading (DL)

AST was added to the mixed solution of dichloromethane:ethanol (1:10, *v*/*v*, 1 mL) [28]. The maximum absorption wavelength of the solution was measured using a UV-Vis spectrophotometer, BioTek, US. The absorbance of different contents of AST solution was detected at the maximum absorption wavelength, and a standard curve was constructed. The nanoparticle solution was lyophilized and dissolved in 1 mL of dichloromethane:ethanol (1:10) mixed solution, the absorbance was measured, and the content of AST was calculated according to the standard curve. DL was obtained using the following equation:

(1) $$\mathrm{DL}=\frac{\mathrm{weight}\;\mathrm{of}\;\mathrm{encapsulated}\;\mathrm{AST}}{\mathrm{weight}\;\mathrm{of}\;\mathrm{total}\;\mathrm{nanoparticles}}\;\times \;100\%$$

### 2.5. Response Surface Methodology (RSM)

The single-factor screening experiments that may affect the DL in the preparation process, including the ratios of drug to carrier and oil phase to water phase, the concentration of PVA, ultrasonic power, and ultrasonic time were conducted. Depending on the single-factor screening experiments, we selected the three factors that have the greatest influence on DL. RSM was adopted in the experimental design. The experiment was performed with three factors and three levels, including 17 runs, and DESIGN-EXPERT 10 software was used for the optimization study.

### 2.6. Particle Size and Zeta Potential

The AST@PLGA@BEV solution was diluted 50 times with ultra-pure water. Then, 1 mL of the diluted solution was added to the potential dish and particle-size dish. The particle size, polydispersity index (PDI), and zeta potential of the AST@PLGA@BEVs were determined using ES90 Nano, Malvern, UK. The samples were diluted with ultrapure-water to appropriate concentration. Each measurement was repeated three times at room temperature.

### 2.7. Transmission Electron Microscopy (TEM)

Transmission electron microscopy experiments used Tecnai 12, Philips, NL. Briefly, 10 μL of AST@PLGA@BEVs were added to dry copper net and stained with 2% phosphotungstic acid. After natural drying, Tecnai 12 was used to observe and then photograph the samples.

### 2.8. Cell Culture

Human colon cancer cells (HT-29) were cultured in 5% CO_2_ cell incubator at 37 °C in RPMI1640 medium supplemented with 1% penicillin–streptomycin and 10% fetal bovine serum. After reaching 90% density, the cells were isolated by trypsin (including 0.25% EDTA) after the experiment.

### 2.9. Anticancer Activity In Vitro

HT-29 survival viability was determined by 3-(4,5-dimethylthiazol-2-yl)-2,5-diphenyltetrazolium bromide (MTT) assay. HT-29 cells were seeded in 96-well plates with 200 μL RPMI1640 for 24 h. Cells were pretreated with the same concentration of AST, AST@PLGA, and AST@PLGA@BEVs for 24 h. Each well was added with 10 μL MTT (5 mg/mL) and incubated in the cell incubator for 4 h. Then, 100 μL DMSO was mixed until the purple crystal dissolved completely under an ordinary optical microscope. The absorbance was detected at 570 nm.

### 2.10. Statistics

Each experiment was performed for at least three repeats. The results were presented as mean ± standard deviation. Statistical mean differences were evaluated using SPSS 25.0. The results were considered statistically significant if *p* < 0.05.

## 3. Results and Discussion

### 3.1. Standard Curve of AST

**Figure 1:**
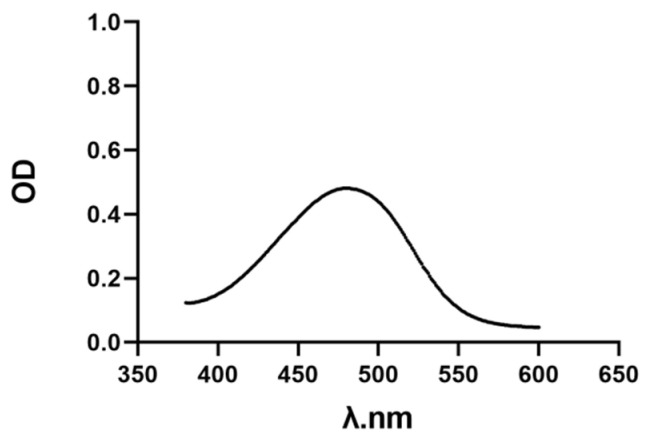
UV-V is spectrum of AST. The absorbance curve of AST was detected by step 2 nm, and the maximum absorption wavelength was found at 480 nm.

**Figure 2:**
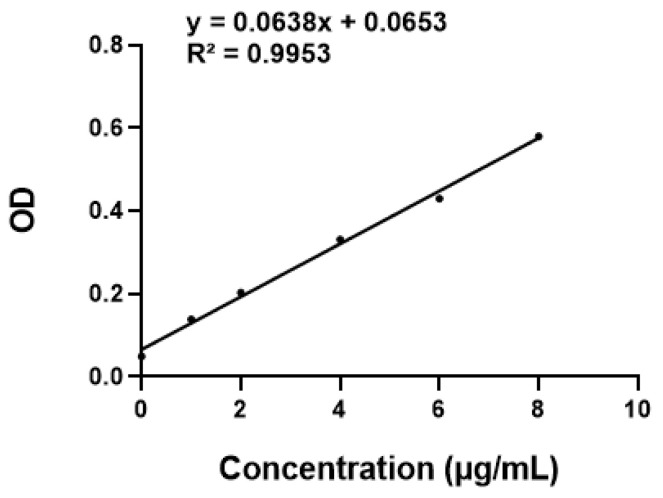
Standard curve of AST.

The ultraviolet spectrum of AST with a certain concentration was scanned by a multifunction microplate detector, and the maximum absorption wavelength of AST at 480 nm was determined (Figure 1). After fitting the absorbance of AST at different concentrations at 480 nm, the linear regression equation of AST was y = 0.0638x + 0.0653 (Figure 2), which was linear for 0–10 μg/mL.

### 3.2. Single Factor Screening

#### 3.2.1. Ratio of Drug to Carrier

The appropriate ratio of drug to carrier can effectively prevent the drug from leaking into the water phase, and the higher DL can be obtained under the condition of higher entrapment efficiency. The results of the ratio of drug to carrier are shown in Table 1. The table shows that when the PLGA ratio increases gradually, the DL of AST@PLGA increases at first and then decreases in size. When the ratio of PLGA is 1:5, the drug cannot be completely encapsulated, and some drug remains free. The free drug precipitates and settles in the aqueous phase, so the distribution is not good and the particle size is unstable. As the PLGA gradually increases to an appropriate proportion, although the overall AST@PLGA weight increases, the carrier can encapsulate more drug, so the DL rate increases. When the drug:carrier is high at 1:20, most PLGA contain only a small amount of AST and more form PLGA nanoparticles, resulting in the waste of carrier and the reduction of drug loading and size [29].

#### 3.2.2. Concentration of PVA

The emulsifying effect is largely affected by the concentration of the emulsifier, and the appropriate concentration of emulsifier can increase the DL. The results of the PVA concentration are shown in Table 2. When the concentration of PVA increased, the DL of AST@PLGA increased initially and then decreased, and the size shows a downward trend. The emulsifying effect of the emulsifier may be linear within a certain concentration. When the content of PVA is 5 mL, the emulsion droplets are unstable, and the formed microspheres aggregate due to the formation of emulsion droplets or uncured microspheres. Large emulsion droplets are difficult to break into small, milk droplets, resulting in a waste of AST and a larger size than that in other concentrations. The higher concentration of PVA indicates higher DL. As the concentration continues to increase, more drug infiltrates the aqueous phase and cannot be encapsulated, showing a free shape; the DL and size also decrease.

#### 3.2.3. Ratio of Oil Phase and Water Phase

The proportion of water phase can affect the content of PVA. The results of the ratio of oil to water are shown in Table 3. The table shows that, with the increase in the ratio of aqueous phase, the DL of AST@PLGA increases initially and then decreases, and the size shows a downward trend, which is similar to that of Section 3.2.2. The content of emulsifier is low because the concentration of the aqueous phase is low, resulting in a poor emulsifying effect and low DL. When the aqueous phase improves, more AST cannot be encapsulated and is free in the aqueous phase. Therefore, the loss of AST becomes higher, and the DL becomes lower.

#### 3.2.4. Ultrasonic Time

Ultrasonic time and power can have a great influence on AST and DL. The results of ultrasound time are shown in Table 4. The table shows that, with the increase in ultrasound time, the DL of AST@PLGA increases initially and then decreases. In the range of 0.5–1 min, the sizes are close to each other, and then decrease. When the ultrasonic time is short, at 0.5 min, the emulsion droplets containing AST and PLGA cannot be completely broken. With the volatilization of dichloromethane, AST gradually precipitates, and the DL decreases. When the ultrasonic time increases, the emulsion droplets are completely broken, and when the time continues to rise, the ultrasonic crusher produces excessive heat, AST is lost by heat, and the DL and size are reduced.

#### 3.2.5. Ultrasonic Power

The results of ultrasonic power are shown in Table 5. The table shows that, with the increase in ultrasonic power, the DL of AST@PLGA increases initially and then decreases. In the range of 0.5–1 min, the sizes are close, and then decrease. The ultrasonic power is small at 50 W due to the low crushing efficiency, which is not conducive to the formation of nanoparticles, and the DL also decreases. The ultrasonic power is extremely high, the heat production is excessive, the AST is lost by heat, and the DL decreases. However, in general, due to the fixed ultrasonic time, the change in DL is small, as is the effect of the ultrasonic power on DL. This condition may be due to the leakage of the AST, which causes a reduction in particle size [30].

### 3.3. RSM

RSM is a technique widely used to optimize various flow processes [31]. According to the results in Section 3.2, three influencing factors, namely the ratio of drug to carrier (A), the ratio of oil to water (B), and ultrasonic time (C) were selected to design 17 rounds of response surface experiments with three factors and three levels. Each group of experiments was repeated three times. The drug loading of AST@PLGA was investigated. The results are shown in Table 6.

The data in the table were analyzed by Design−Expert 10 software, and the effects of various factors on drug loading were fitted by binomial equation. The relevant parameters of model fitting and the analysis results are shown in Table 7.

The DL polynomial model equations are as follows:

DL = 2.164893725 + 0.4592424A + 0.45438B − 1.32725C − 0.010311AB + 0.10342AC + 0.049342BC − 0.021398A^2^ − 0.015783B^2^ − 0.1099913C^2^ (2)

**Figure 3:**
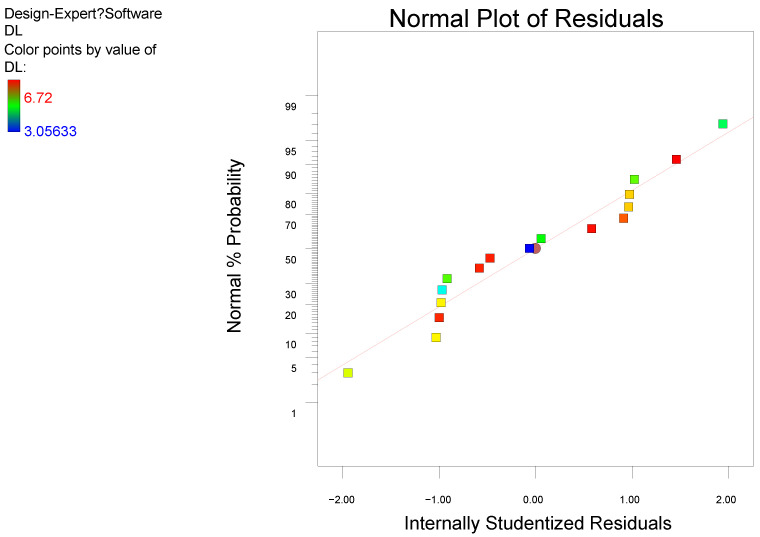
Normal plot of residuals.

The model F-value of 469.54 and *p*-value less than 0.0001 imply that the model is significant. The lack of fit F-value of 1.56 implies that the lack of fit is insignificant, and the normal plot of residuals (Figure 3) may imply that the model fit is good. The results for the DL of AST@PLGA can be predicted and analyzed. The results in Table 7 indicate that the order of the three influencing factors on the DL of AST@PLGA is A > C > B, thereby suggesting that the proportions of drug and carrier have the greatest influence, followed by ultrasonic time and the ratio of oil to water.

**Figure 4:**
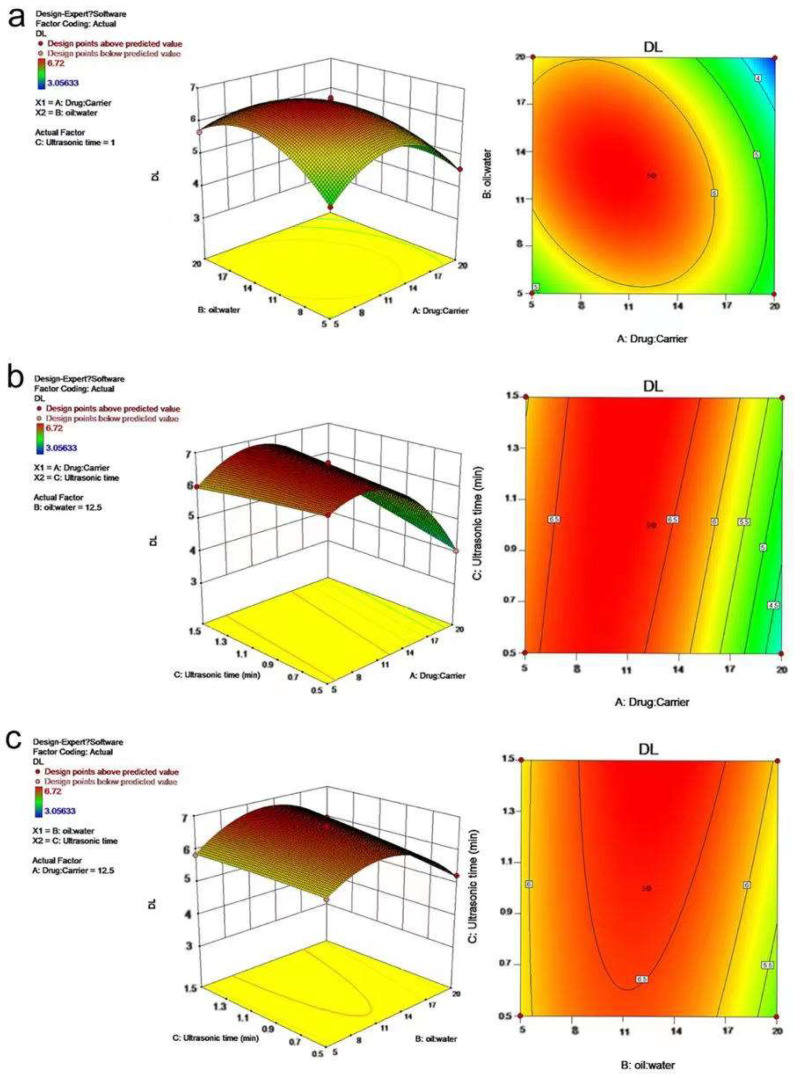
Effects of various factors on DL in AST@PLGA nanoparticles. (**a**) 3D response surface maps and contour map of drug−carrier ratio and oil−water ratio. (**b**) 3D response surface maps and contour map of drug−carrier ratio and ultrasonic time. (**c**) 3D response surface maps and contour map of oil−water ratio and ultrasonic time.

Combined with the above regression model, the response surface map and contour map of the model were drawn by Design−Expert, from which the influence of the interaction of various factors on AST@PLGA drug loading can be analyzed. The results are shown in Figure 4. The steepness of the curve in the 3D response surface map, the density of contours, and the degree of color change in the contour map may reflect the influence of the interaction of the two variables on the DL [32]. The greater steepness of the curve, the denser contours, and the faster color change indicate greater influence on the DL. As shown in Figure 4, the curves of Figure 4a,b are steep, and the steepness of Figure 4c is lower than that of the two former figures. In combination with Table 7, the interaction between AC and AB was extremely significant, whereas the interaction of BC was less than that of the two former combinations. The predicted optimal prescription is an 11.205 drug−to−carrier ratio, 13.079 water−to−oil ratio, and 1.5 min ultrasound time. The predicted DL is 6.824%.

### 3.4. Model Validation

Three batches were prepared according to the best process given by RSM of Section 3.3 to verify whether the process has good repeatability. The three DLs were 6.54 ± 0.02%, 6.89 ± 0.05%, and 6.89 ± 0.22%. Compared with the predicted results, this finding was in line with expectations and has good repeatability. Under this condition, AST@PLGA@BEVEV was prepared according to the method in Section 2.3. The DL is 5.88 ± 0.34%.

### 3.5. Characterization of AST@PLGA@BEVs

**Figure 5:**
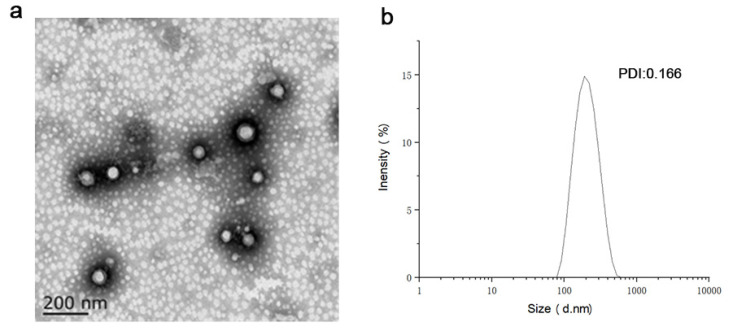
Characterization of AST@PLGA@BEVs. (**a**) Transmission electron microscopic (TEM) images of AST@PLGA@BEVs. (**b**) The size of AST@PLGA@BEVs.

Imaging analysis of particle morphology is an important index to characterize nanomaterials. Commonly used methods include TEM, SEM, and so on. The morphology of the nanoparticles affects the circulation, distribution, cell uptake, and release of drugs in vivo. As shown in Figure 5, the shape of AST@PLGA@BEVEV was spherical, regular, and evenly distributed. The outermost layer is the EV of broccoli, and the inner layer is AST@PLGA. Similar to the results in the currently reported TEM images, it is generally considered that PEV is similar to EV from animals and is quasi-spherical. The outer BEVs are spherical, which is consistent with the results of the current research [33]. AST is an irregular, massive crystal, which more easily enters into the cell after being encapsulated [34]. The size and PDI of nanoparticles are very important characteristics and may affect the toxicity, stability, and biological distribution of the drug delivery [35]. The significance of the zeta potential is that its value is related to stability. The zeta potential is proof of the strength of the mutual repulsion or attraction among the particles. The smaller dispersed particles indicate higher absolute values for the zeta potential and more stable systems [36]. The size of AST@PLGA@BEVs is 191.60 ± 2.23 nm, PDI is 0.166, and the zeta potential is −15.85 ± 0.92 mV. This finding proved that the AST@PLGA@BEVs are uniformly dispersed, stable in aqueous solution, and easier to be absorbed.

### 3.6. Anticancer Activity In Vitro

**Figure 6:**
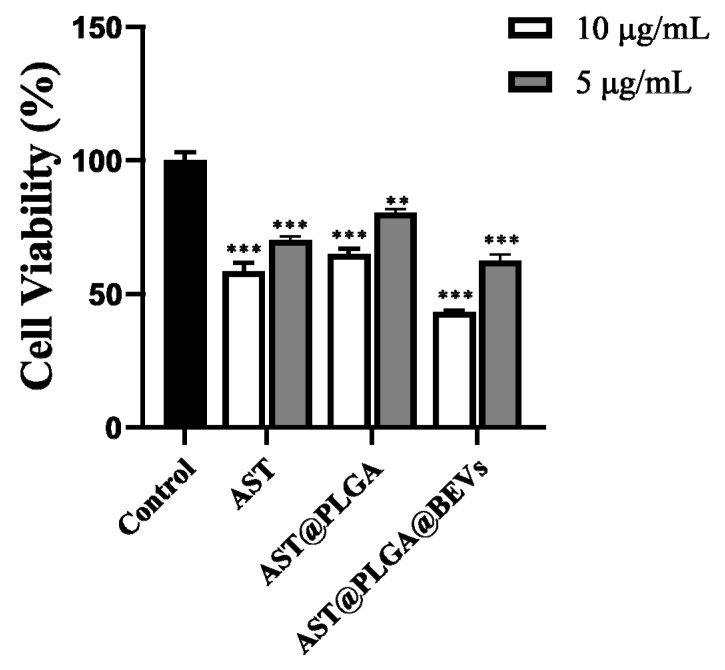
Effect of cell proliferation on HT-29 cells. ** *p* < 0.01; *** *p* < 0.001, vs. Control group.

The anticancer activity in vitro of AST, AST@PLGA, and AST@PLGA@BEVs in HT-29 cells was evaluated using the MTT assay (Figure 6). After HT-29 cells were treated with different concentrations of the three drugs for 24 h, the proliferation of HT-29 cells was inhibited to different degrees, and the inhibitory effect of the same was dependent on concentration. At the same concentration, the cell viability of 10 and 5 μg/mL AST@PLGA@BEVs was 43.29% and 62.41%, respectively. Comparing AST and AST@PLGA, AST changed the cell viability to 58.51% and 70.25%, respectively; AST@PLGA changed the cell viability to 64.90% and 80.45%, respectively; and AST@PLGA@BEV possesses increased anticancer activity. Studies have shown that AST can inhibit the growth of cancer cells by blocking cell cycle progression and promoting apoptosis [37]. The BEVs have anticancer activity and can promote tumor cell apoptosis by specifically targeting the tumor site [38]. PLGA has good biocompatibility and degradability in physiological environments, but PLGA may not enhance the biological activity of AST due to the drug release rate for 24 h. EV extracted from edible plant tissues is one of the safest therapeutic vectors. When BEVs are loaded with AST, they have better anticancer activity, which may be due to the anticancer activity of the BEVs and the combined action of BEVs and AST. The results were similar with a previous study, which integrated the functions of grapefruit-derived vesicles and cargos [39].

## 4. Conclusions

In this study, we successfully loaded AST with broccoli extracellular vesicles and designed RSM with 17 rounds of response surface experiments with three factors and three levels. The optimized DL of AST@PLGA was 6.824%, and the DL of AST@PLGA@BEVs was 5.88%. The size, PDI, and zeta potential of AST@PLGA@BEVs were 191.60 ± 2.23 nm, 0.166, and −15.85 ± 0.92 mV, respectively. TEM results showed that the nanoparticles were circular, regular, and evenly distributed, and AST was successfully loaded into BEVs. In the anticancer experiment in vitro, AST@PLGA@BEVs showed better ability to inhibit the proliferation of HT-29 cells. In our study, the method of loading astaxanthin into broccoli extracellular vesicles is very feasible. Broccoli has high nutritional value, and it is also well known. Based on the vesicles extracted from these natural foods, the construction of a functional food delivery system is more acceptable than other carriers. It provides a new way to deliver functional food, which is meaningful for the research and development of functional food. EVs naturally have the function of loading drugs and can be used to carry hydrophilic and hydrophobic drugs. At the same time, they can also improve the targeting and tissue permeability of the cargos [40]. Compared with EVs from animals, the research on EVs is relatively limited, especially in the field of food. It is worth noting that the method of large-scale extraction should be improved to broaden intensive study and industrial applications.

## Figures and Tables

**Table 1:** Effect of the ratio of drug to carrier on DL.

Drug: Carrier	Size (nm)	DL (%)
1:5	218.95 ± 9.97	4.46 ± 0.05
1:10	161.15 ± 12.23	6.78 ± 0.12
1:20	137.00 ± 2.10	3.69 ± 0.09

**Table 2:** Effect of the concentration of PVA on DL.

PVA (%)	Size (nm)	DL (%)
0.5	210.20 ± 5.43	6.04 ± 0.18
1	161.15 ± 12.23	6.78 ± 0.12
2	134.23 ± 1.72	5.62 ± 0.40

**Table 3:** Effect of the ratio of oil to water on DL.

Oil: Water	Size (nm)	DL (%)
1:5	183.40 ± 3.96	5.81 ± 0.09
1:10	161.15 ± 12.23	6.78 ± 0.12
1:20	149.13 ± 1.66	5.06 ± 0.02

**Table 4:** Effect of ultrasonic time on DL.

Ultrasonic Time (min)	Size (nm)	DL (%)
0.5	162.57 ± 3.41	4.58 ± 0.07
1	161.15 ± 12.23	6.78 ± 0.12
2	137.57 ± 1.32	3.79 ± 0.10

**Table 5:** Effect of ultrasonic power on DL.

Ultrasonic Power (w)	Size (nm)	DL (%)
50	162.10 ± 12.73	6.57 ± 0.45
100	161.15 ± 12.23	6.78 ± 0.12
200	136.10 ± 5.27	6.01 ± 0.15

**Table 6:** The Box–Behnken screening design matrix and responses.

Turn	A	B	C (min)	DL (%)
1	1:12.5	1:5	1.5	5.83 ± 0.07
2	1:12.5	1:12.5	1	6.60 ± 0.03
3	1:12.5	1:12.5	1	6.67 ± 0.12
4	1:20	1:20	1	3.06 ± 0.10
5	1:20	1:12.5	0.5	4.05 ± 0.08
6	1:5	1:20	1	5.67 ± 0.12
7	1:5	1:5	1	4.87 ± 0.16
8	1:20	1:12.5	1.5	5.19 ± 0.10
9	1:12.5	1:12.5	1	6.58 ± 0.05
10	1:12.5	1:12.5	1	6.72 ± 0.15
11	1:5	1:12.5	0.5	6.40 ± 0.17
12	1:12.5	1:20	1.5	5.97 ± 0.16
13	1:5	1:12.5	1.5	5.99 ± 0.07
14	1:20	1:5	1	4.58 ± 0.16
15	1:12.5	1:20	0.5	5.24 ± 0.04
16	1:12.5	1:5	0.5	5.84 ± 0.21
17	1:12.5	1:12.5	1	6.61 ± 0.07

**Table 7:** Statistical analysis of variance for DL in Box–Behnken Design.

Source	Sum of Squares	df	Mean Square	F Value	*p*-Value Prob > F	
Model	17.14	9	1.90	469.54	<0.0001	significant
A	4.61	1	4.61	1136.21	<0.0001	
B	0.18	1	0.18	43.26	0.0003	
C	0.26	1	0.26	64.73	<0.0001	
AB	1.35	1	1.35	331.82	<0.0001	
AC	0.60	1	0.60	148.35	<0.0001	
BC	0.14	1	0.14	33.77	0.0007	
A^2^	6.10	1	6.10	1504.24	<0.0001	
B^2^	3.32	1	3.32	818.36	<0.0001	
C^2^	3.184 × 10^−3^.	1	3.184 × 10^−3^.	0.79	0.4050	
Residual	0.028	7	4.055× 10^−3^			
Lack of Fit	0.015	3	5.108× 10^−3^	1.56	0.3297	not significant
Pure Error	0.013	4	3.266× 10^−3^			
Cor Total	17.17	16				

## Data Availability

Not applicable.

## Sample Availability

Not available.
